# The Ganglionic System

**Published:** 1836-04-01

**Authors:** 


### THE GANGLIONIC SYSTEM.

I. RECHERCHES EXPERIMENTALES SUR LES FONCTIONS DU SYSTEME NERVEUX GANGLIONAIRE, ET SUR LEUR APPLICATION A LA PATHOLOGIE. Par *J. L. Brachet.*

II. DE NERVI SYMPATHETICI HUMANI FABRICA, USU, ET MORBIS COMMENTATIO ANATOMICO-PHYSIOLOGICO-PATHOLOGICA. TABULIS ÆNEIS ET LITHOGRAPHICIS ILLUSTRATA. Auctore *Joh. Frid. Lobstein,* Medicinæ Clinices et Anatomicæ Pathologicæ in Facultate Medica Argentoratensi Professore, &c. &c. &c.

EXPERIMENTAL RESEARCHES ON THE FUNCTIONS OF THE GANGLIONIC NERVOUS SYSTEM, &c. &c. By *J. L. Brachet.* Paris, 1834.

AN ANATOMICAL, PHYSIOLOGICAL, AND PATHOLOGICAL ESSAY ON THE STRUCTURE, USE, AND DISEASES OF THE SYMPATHETIC NERVE. By *J. Frid. Lobstein.*

WE here continue the analysis of the first of the above works from our last number. In preparing the analysis, we happened to alight upon the work of M. Lobstein, on the same subject. The great scarcity of this book, and the high character of its author, will, we trust, be a sufficient apology for our introducing entire a work, which has now been some years published—we do not, however, remember to have seen any analysis of it in any of the periodicals of this country.

M. Brachet next considers the influence of the ganglionic system of nerves on the *action of the stomach.* As in the case of the lungs, he considers the different operations of the stomach, and what influence each species of nerves exercises on it. Three acts depend on the stomach : 1st, the desire of food, or the sensation of hunger; 2dly, the chemico-vital action of the gastric juices on the alimentary mass, or chymification; 3dly, the peristaltic or antiperistaltic action of the stomach on the alimentary mass, so as to present all the parts of it in succession to its chymifying surface, and then to expel it.

And first, with respect to the feeling of hunger ; it is a sensation, obviously a nervous sensation. The want of food is felt, and this feeling or sensation can be transmitted to the brain only by means of nerves. The sensation of uneasiness experienced in the epigastric region shews the seat of it to be there ; the grateful feeling which immediately succeeds the ingestion of aliments, proves that it is the stomach which is the seat of it, and that the other phenomena are but sympathetic, since they disappear even before digestion has commenced. Is it the pneumogastric nerve which transmits the sensation? or the filaments of the trisplanchnic? Our author instituted some experiments in order to solve this problem. In the first he made a spaniel dog fast for twenty-four hours, until it seemed to be devoured with hunger. He then divided the eighth pair below the origin of the recurrent nerves. The animal being untied and left to itself, lay down, and did not seek for any thing to eat, the respiration was free, though a little slow. He then gave it some meat cut into small bits: this he ate, though without seeking for it. The animal continued, till he was unable to swallow more in consequence of the œsophagus being full.

In his next experiment he kept two cabiais fasting for eighteen hours. The animals looked for food in every direction; they raised their heads, and uttered cries indicating the hunger which they felt. He then removed a portion of the two cords of the eighth pair, adopting the precaution of making an opening in the trachea; he then filled their trough with crumb of bread, a kind of food which they had previously appeared to eat with the greatest avidity: they lay down without taking any notice of it; he then put the trough under their nose; they began to eat, but with a sort of indifference. He then removed the trough from them; they did not stir, though they had but six inches to run in order to reach it. He then gave them, in separate pieces, as much bread as they wished, or rather, as much as they were able to eat; for they never stopped, till deglutition became impossible. They eat because the food was before them, and their mouth still relished the taste of it: hunger did not seem to have any influence on their doing so, since they no longer evinced a desire to eat, from the moment the nervi vagi were divided. From these experiments, the author very naturally deduces the conclusion, that the pneumo-gastric nerves are the organs which transmit the sensations of hunger and satiety. In support of this conclusion, he adduces the authority of Valsalva, Baglivi, Legallois, and Professor Dupuy of Alfort, who observed the same results. That such is a function of the pneumogastric nerves, the following considerations render undeniable. Since the animal continues to eat after the stomach is full, even after the œsophagus is gorged with food, it follows, that it is no longer conscious of this fulness, or, in other words, the sensation of satiety is abolished. Now, in these experiments the eighth pair of nerves was divided; it must have been it then which, previously, used to transmit to the animal the sensation of satiety, and which made it refuse food, when no longer necessary. Our author here mentions what he perhaps very fairly conceives to be a case in point—it was a living bicephalous female child, seven months old, which he was just after seeing. It had but two legs, and one abdomen; but its chest was broad and bifurcated superiorly, so as to afford insertions to the four arms in their respective places. The two heads had each a separate and independent life. Though the abdomen was single, our author felt satisfied that there were two stomachs, one for each infant, and gives the following reason for his thinking so:—One of these infants sucks its mother, the other its nurse. He made one of them suck till it was satiated, the other instantly took its nurse's breast, and sucked very greedily. If there were but one stomach for the two children, when it became full, no matter by which of their mouths, both would have deen satisfied; if the one infant was hungry, whilst the other felt satiated, there must be two stomachs. Though the validity of our author's conclusion, with respect to the function of the pneumo-gastric nerves, is not at all affected by the fact of there being only one or of there being two stomachs in this bicephalous monster, still we do not feel warranted in agreeing with him that there must have been two, and our reasons are these; in the first place, there was but one abdomen, of the natural size, not like the chest, which was, as our author tells us, very broad superiorly, in order to afford space for the insertion of the double pair of arms. From this we feel disposed to think that, if there were two stomachs, there would be some indication of it in the enlarged size of the abdomen.

Again, our author's reason for being satisfied that there were two stomachs, appears to us any thing but satisfactory. He says, if there were but one stomach, when that was filled, both would have been satisfied. As we know that the seat of the sensation of satiety is in the brain only, the pneumogastric nerve being but the conductor of the sensation, and not its seat, we can, without much difficulty, conceive that the impression of satiety conveyed by the pneumogastric nerves to one brain, need not necessarily be conveyed to the other. In fact, though there may have been but one digestive apparatus allotted to the monstrosity, still the fact of there being two brains, satisfies us that there were two persons in question, and consequently that what would please, displease, or satisfy one of the young ladies, might not exactly please, displease, or satisfy the other. What we mean to say is, that M. Brachet is not warranted in inferring, *a priori*, that there must necessarily be two stomachs. We remember, of course, the case of Ritta-Christiana, where two stomachs were found in the post-mortem examination. To this, which we acknowledge to be a digression, we have been led, perhaps MISled, by the fond recollection of the many happy hours we have spent over the old school question of "personal identity." The present is however less metaphysical, and more matter of fact, it being neither more nor less than whether *personal individuality*, or *distinction* of person, requires distinct digestive organs. Our province being however medical, though in such a character we may truly say, with the old man in the play, "humani nihil a me alienum puto," we shall, for the present, leave the full consideration of this matter, as to the advantages or disadvantages likely to result from a community of stomachs, in the hands of the political and domestic economist. If partnerships in such a concern were not congenital, but left to the selection of the individual, we are rather inclined to think that certain *persons* in a neighbouring country, whose habits under the existing order of things are not such as to occasion in them much abdominal development, would be disposed to choose for their co-partners in the digestive firm, other *persons* who have hitherto not evinced much *sympathy* with them, whose abdominal protuberance, however, might excite a suspicion, that there was "room for two"—the latter persons, we apprehend, would feel *stomached* at such an arrangement.

MM. Desruelles and Broussais considered the trisplanchnic nerves as the organs which give to the mucous tissue the power of feeling the want of food.—(*Journ. de Sc. Med. July*, 1823.)

M. Desruelles will also have it, that the general phenomena of hunger, and the sudden satisfaction arising from the taking of food, depend on the same nerves. If, our author observes, this gentleman had considered, that these phenomena are nervous, since they cease as soon as the stomach has received the impression of aliment, before this aliment is digested, and long before its nutritive juice has reached the organs, and, above all, if he had observed that these phenomena occur in organs subjected to the influence of the nervous system, he would not have hazarded such an opinion. As a conclusive proof with respect to the agents in the sensation of hunger and satiety, he supposes a very probable case; namely, a person sinking under the effects of inanition, who, exhausted from hunger, sits down, and prefers to die rather than walk a step farther: yet this individual, on being assured that by proceeding to a little distance he shall obtain abundance of food, revives in a manner, and recovers the use of his legs and spirits. He thought himself unable to walk ten steps, and the hope of obtaining food makes him walk ten miles. Here the great sympathetic can have had no share in the thing, as it can have perceived nothing: the brain alone and its nerves were the agents of restoration; they were also the agents of the several phenomena. Like all the other nerves of the senses, the pneumogastric nerves are subject to several modifications of susceptibility, and anomalies of sensation. To them may be attributed those nervous cases of anorexia so often observable, instances of bulimia, pica, &c. In convalescence, after an acute disease, the stomach digests, and yet the individual is always hungry; the reason is, because then all the organs and all the exhausted tissues call for reparative materials, demand them from the stomach, whose business it is to send them, and so keep up in this organ the continued sensation of want, which, in this case, is but sympathetic. This is not the only sensation which the pneumo-gastric nerves are commissioned to receive—they perform an important part in the exhibition of medicinal substances. It is they which receive the impression of the medicine, and which transmit it to the encephalon. If a narcotic be taken, its action on the encephalon is perceived almost instantaneously, and long before the medicine has been digested and absorbed. This transmission can take place only by means of the eighth pair, which receives the impression, and transmits it immediately to the brain. For the purpose of comparison, the author administered six grains of opium to two dogs of the same size, having previously divided the pneumo-gastric nerves in one of them. The dog which had not been operated on soon experienced and evinced all the symptoms of complete narcotism, whilst the other lay down quietly, and showed no other symptoms than the difficulty of breathing natural to the operation.

Another fact worthy of remark is, that nux vomica, which so rapidly produces the signs of poisoning, produces no effect as soon as the eighth pair of nerves has been divided. You may give a double, or treble dose of this substance—poisoning will not take place in the first instance; it evinces itself only at a much later period and with less intensity, unless the dose be very large. Hence it is evident that *strychnine* acts on the cerebral nervous system, since it exerts no action, when the nerves with which it is related are divided, and that it acts only when it is carried by absorption to the brain. The same observation may be applied to all medicinal substances. In whatever dose emetics and purgatives be given to dogs in whom the nervi vagi have been divided, no effect is produced; the brain perceives nothing—there is no reaction. The remedy produces no effect, unless it be absorbed, and act directly on the contractile fibre. Every one knows that, when the encephalon is the seat of any affection which retards its functions, the action of remedies is much less efficacious, and that then double or treble doses are required to produce the same effects. Why is this? Merely because the diminished cerebral action renders the nervi vagi much less susceptible of impression. Incontestable proofs are every day furnished of this circumstance, in cases of apoplexy, cerebral inflammation, and effusion, &c.

It is also by the pneumogastric nerves that we can explain the extraordinary effects of the re-action of the stomach on the encephalon, sympathetic effects, which perform a great part in diseases, and to which, perhaps, too much has been conceded in latter times. Let us only observe what occurs in intoxication, and we will then see the effect of the action of the nervus vagus, much more than that of the absorption of the spirituous liquor. Intoxication is produced before absorption can have taken place ; as a proof of this, we may adduce the almost sudden effect of liquid ammonia in a drunken man. Let him take but a few drops of this alkali in a glass of sugar and water, and the state of intoxication disappears almost suddenly, and as if by enchantment. The wine, then, must have acted only on the nervi vagi ; the ammonia, too, must have directed its action to them. This is the only mode of accounting for these phenomena. We constantly see the violent headaches produced by bad digestion, as also the intolerable megrims occasioned by collections of bile, or indigestible substances in the stomach, which megrims can only be removed by dislodging the offending matter by vomiting, &c.

Our author next considers the peristaltic and anti-peristaltic action of the stomach. After adverting to the discoveries of the distinguished physiologists who directed their researches to this subject, particularly Blainville, Brodie, Legallois, Wilson Philip, &c. he remarks that, whilst it must be admitted that these distinguished experimenters have added much to our knowledge on this interesting subject, still that as many different opinions have been started on the question at issue as there have been experimenters—each of them endeavouring to explain whatever was found to contradict the experiments and opinions of the rest. So that, whilst the nervous influence was admitted on all hands, its mode of action was misunderstood, in consequence of the general way in which they viewed the question. All their researches were directed to ascertain the influence of the nerves on digestion, and, as their experiments were confined exclusively to the pneumogastric, as they were the only nerves which they could reach with their instruments, every thing was referred to these nerves, no notice being taken of the other nerves which the stomach receives, and, consequently, no account being taken of the influence which the latter may still continue to exercise. As the process of digestion consists of several acts, and as each of these may be subjected to various influences, they could not appreciate these special influences, inasmuch as they always considered digestion en masse ; so that the influence of one nerve on one of these acts became the nervous influence on digestion. In order to proceed more methodically, it becomes necessary, then, to attend —1st, to the different species of nerves which the stomach receives ; 2dly, to the different acts which constitute digestion ; 3dly, to the influence of each species of nerves on each of these acts. It was only by proceeding in this way, that one could hope for a satisfactory solution. As has been already mentioned, the stomach receives nerves from two sources—from the cerebral system and the ganglionic system : the nervi vagi are the cerebral nerves of the stomach, and the nerves of the stomachic coronary plexus are its ganglionic nerves. It is very important not to lose sight of these two sorts of nerves, if we would obtain positive data with respect to the nervous influence on digestion. The acts of the stomach on the alimentary mass may be reduced to two principal heads. These are—1st, the peristaltic and antiperistaltic movement, by which the alimentary mass is moved to and fro, and then expelled from the stomach; 2dly, the secretion or exhalation of the juices or liquids, whose chemico-vital action on this mass constitutes chymification. Our author now proceeds to detail some of the facts connected with this subject, as it will afterwards be easy to deduce from them the most satisfactory explanation. His object not being, like that of some of his predecessors, to ascertain whether the nerves could, after being simply divided, transmit the nervous influence, he premises that, in nearly all his experiments, he took care to ensure loss of substance in the nerves, and to cut off every kind of connexion or direct communication.

*Experiment* 54. After making a dog to fast for twenty-four hours, he made him eat a large quantity of meat; immediately after, he divided the eighth pair. Ten hours after, he opened the animal, when he found the stomach distended, still containing all the food that had been taken. All the small intestine was empty. The stomach, which was opened with great care, presented the alimentary mass but little changed, except on the surface, which was covered with a thin layer of greyish and mucous chyme; all the inner parts of the alimentary substances retained the appearances peculiar to each.

*Experiment* 55. For the purpose of comparison, he killed a dog of the same size, which he had also made to fast and eat at the same time as the preceding dog. The stomach was empty, and contained nothing but some greyish mucus. The small intestine was likewise empty; the large intestine alone contained the natural residue of digestion.

*Experiment* 56. After having made a dog to fast, which was of the same size as the preceding, he made him eat at the same hour. He then made an incision on both sides of the neck, and exposed the nervi vagi without cutting them. He then let the animal lie for ten hours, as in the preceding cases. On opening the body, he found, as before, the stomach and small intestine quite empty, and the large intestine full.

*Experiment* 57. He repeated the three preceding experiments on three young dogs of the same size, with this only difference, that he opened them six hours after they had eaten. In the first, that whose nervi vagi were divided, the stomach contained all the food that had been taken; its surface was covered merely with a very thin layer of chyme, much more marked towards the pylorus than towards the cardia. In the second, that which underwent no operation, digestion was complete. In the third, digestion was not completed; the stomach still contained a great quantity of clear grey chyme, of the consistence of an almost liquid pap, in which were found some remains of tendon and bones. The duodenum and small intestine contained a part of the chyme, and the lacteal vessels were filled with chyle.

He repeated these three experiments several times. The two first always presented the same degree of advancement in digestion, whilst, in the third, he obtained results extremely varied. Sometimes digestion was as complete as in the second; at other times, it was not more advanced than in the first.

*Experiment* 58. Immediately after having made a dog eat, he made on the sides of the chest two deep and large incisions; he then, from time to time, irritated the wounds with his fingers, and six hours afterwards he opened him. Digestion was nearly complete; there only remained in the stomach some slight traces of chyme, and some portions of tendon. The chyliferous vessels were gorged with their white juice; the duodenum was nearly empty, as were the small intestines; the large intestine contained nearly all the fæcal matter.

As it was important to verify the results of this experiment, he repeated it on several dogs, varying the seat of the wound; he never obtained precisely the same results. The advancement in the process of digestion was always in the inverse ratio of the severity of the injury inflicted.

*Experiment* 59. After a fast of 15 days, and immediately after dividing the eighth pair, he made an old horse eat a quantity of oats. He killed him in eight hours after, by introducing some bubbles of air into the jugular. The stomach was distended with all the oats he had eaten. The grains at the surface were half digested, pulpy, and readily bruised between the fingers. The greater number, those occupying the centre, were softened, but not changed. The lacteal vessels contained no chyle.

*Experiment* 61. Whilst sojourning for a time in the country, a horse in his neighbourhood happened to fall from a height, and broke his thigh. It being the usage to sacrifice the animal in such cases, he determined to take advantage of the occurrence. He let the animal fast for 10 or 12 hours, and then brought him some oats, which he ate as if he were quite well. Immediately after, the two pneumogastric nerves were divided. Eight hours after he killed him, by introducing air into the jugular vein. The intestines were empty, except the large intestine, which contained some remains of oats, evidently belonging to preceding digestions. The stomach was distended, and contained all the oats which had been eaten. At the surface, chymification was complete to the depth of two or three lines towards the cardia, and of four or five lines towards the pylorus. Most of the grains were softened and pulpy; some of the oat-husks were empty; a greyish, chyme-like matter was interposed between the grains, whilst those of the interior of the alimentary mass were sound and intact, though a little swollen.

*Experiment* 63. After having suffered a rabbit to fast for 15 hours, he made it eat several leaves of sorrel; and, immediately after, a portion of the two nervi vagi was cut out on each side. Notwithstanding some efforts at vomiting, the animal threw up nothing. He was killed seven hours after. The intestines were empty, and the chyliferous vessels were not apparent. The stomach was distended by the leaves it had swallowed, which, on the surface, presented an evident appearance of alteration, particularly towards the pylorus, where they were reduced to a pulpy homogeneous mass, of a greenish-grey colour, and a particular odour; internally, they presented only a very minute division, without any apparent alteration, and they retained their natural odour.

*Experiment* 70. In another experiment, our author removed a small portion of the posterior lobes of the brain, and a portion of the medulla oblongata, as far as below the origin of the eighth pair, in a young dog, immediately after having made him eat two ounces of boiled meat. He was able to keep the dog alive for five hours. The stomach contained all the food. This was intact on the side towards the cardia, and in all the interior of the mass which it formed; but towards the pylorus it was more moist, and had already undergone a manifest change on its surface.

We shall not, for the present, detail any further experiments, but shall present to our readers the conclusions deduced by our author. Before stating them, however, he lays it down, that the digestion of aliments taken into the stomach requires for its completion—1st, the action of a gastric juice, poured out from all parts of the stomach on them; 2dly, a peristaltic alternating motion, which presents the aliments successively to the parietes of the stomach, for the purpose of impregnating them with this gastric juice, by which motion, also, they are expelled from this viscus. As his experiments, as yet, have been only on the pneumo-gastric nerves, he can, for the present, appreciate the influence of these nerves only on digestion.

In all the animals in which he divided this nerve, with loss of substance, at the moment they had just eaten, he invariably found the stomach filled with aliments, and the intestine empty. The same effect was obtained by acting on the brain, either by removing this viscus, or by the injection of narcotics. The surface of the alimentary bolus was well impregnated with the gastric juices, and even partly reduced to chyme; but this is not sufficient—it is necessary that the aliments be agitated to and fro, and pressed in different directions by undulatory or peristaltic movements, by which every part of these aliments may be successively presented to the internal surface of the stomach, to produce a more intimate combination with the gastric fluids, and in order that their action may be in some measure more vital, as they are poured out at the moment they are formed. It is further necessary, according as chymification is effected, that this motion should expel the aliments already digested or chymified, in order to make way for others. Now this motion of the stomach is owing to the contraction of the muscular fibres extended over its parietes, and this contraction is subject to nervous influence. Since the substances swallowed could not be expelled from the stomach after the operation, it follows that there must have been an abolition of this contraction, or, in other words, a paralysis of the muscular plane. The food may accumulate in the stomach, because it is propelled by the action of the pharynx and upper part of the œsophagus which are not paralysed; it may accumulate beyond measure, even so as to regurgitate into the œsophagus, as we have already seen, but it goes no farther, as the stomach is not empty. As it may happen that a small quantity is occasionally found in the duodenum, we would be wrong in supposing that the stomach propelled it there; it came there only by a mechanical impulsion; it was pushed on by the last portions of food introduced into the stomach, which were themselves propelled by the pharynx and œsophagus. The stomach merely performed the part of an inert tube, without taking any active part in the matter. What, then, does the stomach want, in order to be enabled to continue the agitation and expulsion of the food, as before? The nervi vagi are the only nerves that have been divided; these nerves, therefore, are destined to keep up the motions of the stomach; it is their office to carry to its muscular layer the nervous action, under the influence of which it contracts.

Our author here anticipates an objection which may be made to him, namely, that frequent efforts to vomit have been observed to take place after the eighth pair has been divided, particularly if the division has been attended with loss of substance. On some rare occasions, even a small portion of the stomach's contents has been actually rejected. To this he replies, that this is only regurgitation, not vomiting; the only portion rejected is that which occupied the upper part of the œsophagus, or even of the pharynx, which organs were not paralyzed; the stomach is not at all emptied. Nay, he says he will go farther, and not deny the possibility, or even the actual occurrence of vomiting. It will be objected, he says, that since there is antiperistaltic motion, there cannot be paralysis. To this he replies, that if the primary cause arises from a particular state of the stomach, the material or acting cause lies entirely in the respiratory muscles, among which are those of the abdomen. The latter, and the diaphragm particularly, contract with violence, and the stomach, immoveable and inactive, sustains their efforts; it in fact becomes here the bladder of M. Majendie, in his splendid experiments on vomiting; so that the portion of food which has been voided in these cases was forced out by expression.

"I even admit," says our author, "that the stomach has contracted antiperistaltically; this contraction involves nothing that contradicts my opinion. A nerve is divided, the muscles to which it goes are paralysed. Yet we see these muscles agitated convulsively every time the portion of the nerve separated from the nervous centre is stimulated in any way whatever. The nervi vagi, whose cut extremity is connected with parts of a different kind, meet in these parts a cause of excitation, and the slightest movements of the animal produce a friction still more irritating. The nerve thus stimulated, acts irregularly on the stomach; thence arises vomiting, which is the real convulsion of this organ. This is so true, that if the stimulation be kept up artificially in a regular, constant, and uniform manner, for instance, by means of the galvanic pile, the motion will become regular, and the food will be expelled from the stomach nearly as well as in the animal under ordinary circumstances, as has been proved by the experiments of Dr. Wilson Philip and others...... Galvanism acts here, as it does on all nerves which go to muscles; it takes the place of the nervous influence, so as to stimulate the muscular fibre, cause it to contract, and to agitate the alimentary mass, in order that chymification may be effected on all points of it, and the expulsion of the chyme may take place. There its action is limited; it has no chemical effect on the alimentary bolus for its chymification, as had been supposed."

Our author, in order to ascertain the just value and precise effects of galvanism on the process of digestion, instituted some experiments similar to those made by Wilson Philip, and others. The conclusion to which he arrived was, that the manner in which the galvanic fluid acted, differed not from that of any other mechanical and irritating agent, which might have excited the contractions of the muscular fibre of the stomach by stimulating the eighth pair of nerves. Those artificial contractions produced the same effect as those which are natural; they tossed the alimentary mass to and fro, and presented the different parts of it to the surface of the stomach, so that these parts might become impregnated with the gastric juice; and, finally, they expelled the parts which had undergone chymification from this viscus—in a word, the action of galvanism on all the other muscles of the animal economy should make us conclude, by analogy, that this action is the same everywhere, and that the muscular plane of the stomach is not exempted from the general law of excitation, which it produces on all the muscles.

Our author next notices an opinion of M. Majendie with respect to the influence of the eighth pair in digestion. M. M. thinks that the division of this nerve acts but secondarily on the stomach ; that its primary action is on the lungs, and that it is only in consequence of the disturbance produced in the respiration that digestion becomes arrested or suspended. He founds his opinion on this circumstance, that digestion is deranged by dividing this nerve only when the division is made in the neck, above the origin of the branches which it gives off to the pulmonary plexus, whilst on cutting both these nerves in the chest below the branches which go to the lungs, the food introduced into the stomach becomes changed into chyme, and ultimately furnishes an abundance of chyle. Our author attempted to repeat this experiment several times to ascertain the correctness of M. Majendie's conclusions—in one experiment, he imagined that he succeeded ; in that, he made a dog, on which he operated, eat a small quantity of dressed meat, he let him rest for half an hour, and having killed him six hours after, he found the process of digestion considerably advanced. This of course tended to upset all his conclusions with respect to the mode of action of the nervi vagi on the stomach. He investigated the matter with all possible care. He ascertained that, in consequence of the plexiform arrangement of the pneumo-gastric nerves on the œsophagus, it was impossible completely to divide, unless by entirely dividing the œsophagus; so that the experiment, which had staggered him at first, could not lead to any positive result.

The author in recapitulation states, that the conclusion to which his experiments have led him, is, that the pneumo-gastric nerves are the agents which keep up the contractile action of the muscular plane of the stomach, and that their division occasions its paralysis. As these nerves arise from the encephalon, and serve only to transmit its influence, the result is, that it is on the brain itself that the contractions of the stomach depend. One is no doubt struck with the extraordinary circumstance of cerebral nerves bringing to organs an influence independent of the will. To this we say that the nervus vagus is not the only nerve that does so ; it may also be remarked, that this nerve does not reach the stomach till after it has formed a long plexus around the œsophagus. Might not the object of this gangliform plexus be to weaken the nervous action of the encephalon, says our author, or rather to arrest the effect of the will ?

After adverting to the reciprocal influences of the stomach and brain, the vehicle of this reciprocity being the pneumo-gastric nerves, he makes an observation regarding the separate functions of the nerves of sensation and the nerves of motion. M. Majendie, in testing this theory by ingenious experiments and conclusive facts, has made considerable progress in the study of the functions of the nervous system. The pneumo-gastric nerves resemble the spinal nerves in their origin. Some filaments of their roots descend in oblique fibres from the edge of the calamus scriptorius, which belongs to the chord of sensation; the other filaments arise from the corpus olivare which is continuous with the chord of motion. According to this, the pneumo-gastric nerve must discharge the double function of a nerve of sensation, a nerve of motion, and, in fact, this is what may be seen in the lungs and stomach. In the heart it appears destined only to transmit the pathological and moral sensations, to establish a more direct communication between the two orders of life; but in the two other organs, it is at once a nerve of sensation and a nerve of motion. With respect to the lungs, on the one hand it receives and transmits the want of respiring, of expectorating the mucus collected in too great quantity, and of expelling the foreign bodies which may be introduced into them: on the other hand, it gives to the contractile fibres of the bronchi the faculty of contracting, when this has become necessary for the respiration as for the different expectorations. With respect to the stomach, it is the organ of hunger and satiety, the sensation of which it transmits to the brain; it communicates also to the muscular tunic the nervous influence on which its contractions depend. Thus this nerve, by its functions, as by its origin, confirms the theory of M. Majendie, at the same time that it receives from it a direct application which may add, if possible, more confidence to the different opinions expressed in this work in relation to its functions. If, however, the theory of MM. Majendie and Bell, says our author, were false, (we own that we were surprised at his not alluding to Bell long before,) facts would still remain the same, and the pneumo-gastric nerve would still continue to discharge its functions.

M. Brachet now comes to the subject of *chymification*, or that chemico-vital act by which the food is impregnated with the gastric fluids, in order to be converted into chyme. As this act depends entirely on the exhalation or secretion of a particular liquid poured out on the surface of the stomach, which liquid is called the gastric juice, the next question is, does the formation of this liquid depend on the influence of the eighth pair? The experiments of our author, as well those which we have mentioned in this analysis, as others which we shall not notice, led him to the result, that the eighth pair has no influence whatever, either on exhalation and secretion, or on the absorption which takes place in the stomach. Hence he arrives, by a negative argument no doubt, or, as he calls it, par voie d'exclusion, at the conclusion that these two gastric operations depend on the ganglionic nervous system. This consequence is fairly deduced, no doubt, as we cannot point out any other nerves received by the stomach, except those already mentioned, and as these acts must be performed under some nervous influence or other. Direct and positive experiments on the ganglionic nerves our author frequently attempted, but to no purpose. So that it was only after the results which he attained from his experiments on the eighth pair, that he was able to deduce any conclusions, which fortunately were of the highest consequence, inasmuch as they shew that the pneumo-gastric nerve perceives the want of food, and transmits to the stomach its contractile power, and the great sympathetic nerve only presides over the exhalation and secretion of the gastric juices, and the absorption of the fluids taken into the stomach.

We shall now take a rapid view of the influence of the two nervous systems over the functions of the other parts of the intestinal tube. We shall no longer trouble the reader with a detail of the author's experiments on this part of his subject, but shall come at once to the results. On dividing the eighth pair, some hours after taking food, he concluded that the duodenum and upper portion of the small intestine were paralysed, inasmuch as the alimentary substances could not go further, and because, several hours after the operation, these aliments were not found in the same place as in animals opened at the same hour, without the nerves having been divided. This influence, however, he found did not extend through the entire length of the small intestine, since the lower part of this intestine was able to empty itself, and the food contained within it at the time of the operation, reached the last intestine well digested. One of the author's experiments proved that this part of the small intestine was under the influence of the spinal cord, since the division of this, at the lower part of the back, allowed the chyme to accumulate in its cavity and in the large intestine. This was a matter to be anticipated, seeing that these organs receive nerves from this portion of the cerebro-spinal system. It would be desirable to be able to establish with precision the point where the influence of the nervus vagus ends, and where the influence of the spinal nerves commences; in a word, what is the line of demarcation between these two species of cerebral nerves. This is possible, and probably very readily determined; this, however, our author has not attempted. The muscular layer of the small intestine receives then from the cerebro-spinal system the nervous influence which serves as its stimulus; it is dependent then on this system, with respect to its contractile action.

In some of our author's experiments not detailed in this analysis, it was ascertained that, notwithstanding the division of the eighth pair and of the spinal marrow, the stagnation of the chyme in the small intestine did not prevent absorption from going on, since the lacteal vessels were always found full of chyle, and the quantity of liquid injected into the small intestines, in one of his experiments, was found to be perceptibly diminished. The results thus obtained were conformable to those of M. Majendie, in his beautiful experiments on venous absorption. It concerns not the present inquiry, as our author very fairly observes, whether this absorption takes place by veins, or by the chyliferous vessels exclusively, or by both combined. All that is essential for the present purpose, is to know that this function of absorption continues, and that the division of the cerebral nerves which go to this portion of the intestine does not destroy it. Now this function, continuing, notwithstanding the separation of the intestine from the cerebro-spinal centre, cannot be dependent on this system. It takes place, as in all other animated beings, more particularly as in vegetables, under the sole influence of the ganglionic system of nerves.

With respect to intestinal exhalation and secretion, these certainly continued, since the chyme presented, in the experiments wherein the intestinal nerves were divided, the same changes as in the animals killed for the purpose of comparison. As in the stomach and every where else, these two functions depend on the ganglionic system; consequently, the division of the cerebral nerves cannot influence them directly.

*Influence of the Nervous System on the large Intestine.* Since the eighth pair ceases to send branches, and to transmit its influence to the lower part of the small intestine, *a fortiori*, its influence disappears on the lower part of the large intestine, which receives its cerebral nerves only from the spinal cord. Now these latter nerves act as the preceding, by directing their stimulating influence towards muscular contraction. Cut the spinal cord in the midst of the lumbar vertebræ; by paralysing the cauda equina the rectum also becomes paralysed, and the fæces accumulate in this intestine. Our author divided the spinal marrow between the third and fourth lumbar vertebræ in a cur dog—the cauda equina was paralysed. He kept it for two days, during which it ate nearly as much as usual. It passed neither fæces nor urine. The large intestine was distended with an enormous quantity of fæces. The bladder also was distended with urine. He repeated the same experiment on a cat six weeks old, which he kept for eight days. During this time the cat took soup and milk as before. The first thirty-six hours it had no evacuation—its abdomen was prodigiously distended—it then began to pass from time to time a small jet of urine, which was renewed every hour. On the fourth day it voided some fæces, and then continued for three days without passing any. On killing it he found the bladder enormously distended, though it had passed urine an hour before. All the large intestine was filled with fæces, and yet the cat had passed a considerable quantity the day before. In these two experiments we find paralysis of the bladder and large intestine occasioned by the division of the lower part of the spinal marrow. It may be said, with respect to the experiment on the cat, that it passed urine after thirty-six hours, and voided some fæces at the end of four days; consequently, neither the bladder nor large intestine were paralysed, and consequently their contraction was independent of the nervous action of the spinal marrow. The fact is true, but the consequence is false;—the cat passed urine and fæces; but this action was independent of the contraction of the bladder and rectum; the animal passed only in consequence of repletion. Both these organs, being enormously distended, would not admit of greater extension; consequently every additional portion must propel that which was anterior to it, and so cause it to pass on. The contractions of the abdominal muscles also must have contributed to expel the contents of the bladder and rectum by pressing them in every direction. As a proof that the bladder and rectum had nothing to do with the excretion, they were never emptied. The bladder was prodigiously distended, though the cat had passed urine a few minutes before it was killed: the large intestine was completely filled, though fæces had been passed the day before.

Our author here mentions a case of a countryman brought into the Hôtel Dieu, in consequence of a fall from a high tree, by which the vertebral column was fractured. On his admission, there was mobility and displacement in the part fractured. The lower extremities were completely paralysed, as were also the rectum and bladder. The patient experienced a sense of tightness arising from the fulness of the abdomen; but he felt no necessity either to go to stool or to void urine. Two pints of urine were drawn off by the catheter. The elasticity of the bladder, and contractions of the abdomen, at first caused the fluid to flow out in a strong jet, which however soon ceased, though the bladder still contained a great quantity of urine, for the discharge of which it became necessary to make pressure over the hypogastrium. After three or four days the introduction of the catheter became very difficult;—this was found to be occasioned by an accumulation of hardened fæces in the rectum, which it became necessary to remove with the finger, as a lavement could not be admitted. This fact proves that the muscular contraction of the rectum and bladder is dependent on the cerebro-spinal nervous system. It also proves that the sensation peculiar to these organs—that sensation by which the encephalon is apprised of the fulness of the rectum and bladder, and of the necessity of emptying them, is destroyed by disorganization of the spinal cord, since the patient had no sensation of this necessity, and as he made no effort to satisfy it. This circumstance shews that here, as in all the sensations of necessity, it is the cerebro-spinal which perceives them, and which is their agent or organ.

In support of this opinion our author cites the following case. M. de M——, who had been a soldier for several years in 1814 and 15, experienced some rheumatic pain affecting principally the lumbar region—in 1816, he fell from his horse. The pains left the lumbar portion of the vertebral column. The lower extremities became the seat of a painful sense of formication; one of the spinous processes of the lumbar vertebræ projected; the formication was changed into a sense of numbness, and by degrees paralysis of the lower extremities and of the lower fourth of the abomen became complete. Bleeding, both local and general, moxas, cauteries, setons, mineral waters, nux vomica, drastic purgatives, &c. all proved of no use; sensation and motion were abolished. For the last eight years this paraplegia has not changed either for the better, or otherwise. M. de M. never experiences the sensation of a necessity of evacuating his bowels, still he goes to stool and passes his urine. He is apprised of the necessity of doing so by the distention of the abdomen. He then forcibly contracts the diaphragm and abdominal muscles, and assists their action by pressing with his hands on the umbilical and hypogastric regions; the fæces and urine escape, but without causing any sensation to the patient, who never perceives it except by the diminution in the size of the abdomen. Since he has been in this state, he has had two children. The spermatic secretion goes on perfectly well; erection takes place, and the ejaculation of the semen supervenes; but it passes off without any shock, and without producing that keen and voluptuous sensation, which usually accompanies it. He has had three attacks of gonorrhœa during a long absence from his wife; the mucus flowed in abundance, and the passage of the urine caused no pain: they each lasted about six weeks. In October of 1825, M. de M. had a very severe attack of enteritis. The intestinal exhalation and secretion were increased, and diarrhœa came on. The evacuations were accompanied with colicky pains; but their passing was not at all felt, though the patient had hæmorrhoids in a very inflamed state. In this case M. Brachet conceives it very probable that the fall produced myelitis, or at least spinal meningitis, the severity of which was increased by the rheumatic disposition seated in this region. The disorganization or compression of the cord was the sequence, and paraplegia the consequence of this. Whatever be the rationale of the matter, the fact itself proves this:—1st, that the alteration of the spinal cord paralysed not only the lower extremities, but also the organs to which it sends nerves, as the rectum and bladder; 2dly, that the paralysis was not confined to the power of motion, as it extended also to sensation, the patient not feeling either the necessity for evacuating his bowels, nor passing of the excretions; 3dly, that this paralysis interferes not with the secretions and exhalations, since there was posteriorly copious diarrhœa, and anteriorly three gonorrhœas. Thus the abolition of the functions of the sensorial nerves abolished those acts only which were within their province—muscular contraction and the sensation of necessity—and had no influence over those acts which are foreign to them, namely, the acts of exhalation and secretion, since these continued. Accordingly, these two acts depend essentially on the influence of the ganglionic system.

Absorption took place in the rectum, for, during the enteritis with which this patient was attacked, small lavements of decoction of bran, and ten drops of the liquid laudanum of Sydenham were administered; they were, for the most part, retained, particularly when the purging stopped, and they were absorbed. Thus the rectum, like the stomach, is subject, on the one hand, to the cerebro-spinal influence for the sensation of necessity, and for its contractions; and, on the other hand, to the ganglionic influence for exhalation, secretion, and absorption.

Our author next introduces some experiments, to establish the functions of the two nervous systems in generation. Without detailing the experiments, we shall merely state the result: and first with respect to the *male*—he ascertained that the secretion of semen is totally independent of the influence of the cerebro-spinal system, inasmuch as he found this secretion to go on, notwithstanding the lower part of the spinal marrow was divided; so that, by analogical, and indirect or negative arguments, it may be fairly concluded that this, as well as all the other secretions, is solely under the influence of the nervous system. By experiments on female animals, and by observing the effects of disease of the spinal cord in women, he arrived at the conclusion, that the cerebro-spinal system was not essential to conception.

We shall proceed to his observations on the influence of the ganglionic system of nerves on the Secretions. The secretions are essentially connected with the functions of assimilation; they are found in all organized beings, both animal and vegetable. As the latter are destitute of a cerebral nervous system, and, as they evidently secrete, it cannot be disputed that these functions depend on the ganglionic nervous system, this being the only system they have. By analogy, we may conclude that this is, in animals, the source of the action of their secreting organs.

"There (says our author) what we have to say on the secretions should cease, since our end is attained, and since we have already seen, in the preceding experiments, 1st, that the pulmonary mucous membrane secreted mucus, notwithstanding the division of the cerebral nerves which go to the lungs; 2dly, that the gastro-intestinal mucous membrane performs the same secretion, notwithstanding the division of the par vagum and spinal cord; 3dly, that the genito-urinary mucous membrane secretes mucus, though artificially or pathologically paralysed; and, 4thly, that the testicles secrete semen, though deprived of the influence of the cerebral nerves."

The different and contradictory opinions entertained by physiologists regarding the subject of secretion, and the necessity for the integrity of nerves which go to an organ, in order to its normal secretion, our author refers to the circumstance, of these several experimentalists not making the necessary distinctions between the two orders of nerves.

Our author here details a great number of experiments, undertaken for the purpose of ascertaining which system of nerves is concerned in secretion. The results to which he arrives are, that "the urinary secretion, and, indeed, all the secretions, are performed under the direct and positive influence of the ganglionic nervous system; that it is, in a word, this system, which, by means of its numerous branches, conveys into the glands the mode of innervation necessary for the due fulfilment of their functions."

"I am (he says) convinced that, if it were possible to withdraw for a time a gland from the action of the ganglionic nerves, it would not only cease to secrete its usual fluid, so as to allow nothing but blood to escape into its excretory ducts, but the organ would cease to be nourished, would become impregnated with the blood conveyed to it, but would no longer change it either into a new liquid, or into its own substance."

*Exhalation* is also dependent on the ganglionic nervous system. This cannot be proved, to be sure, by direct experiment, as the various exhalations take place only from extended surfaces, and the membranes which are the organs of them do not receive a nerve, or a fasciculus of nerves, but receive, at every point of their adhering surface, thousands of imperceptible nervous branches. Pathology every day affords instances of this. In paraplegia, or hemiplegia, the limb or part paralysed is found to transpire not less than the healthy limb. Whence, as the organs of exhalation no longer receive any influence from the cerebral nervous system, the function in question must recognize some other, namely, the ganglionic nervous system.

We have now followed our author through the principal parts of his book. We give him great credit for the zeal and industry evinced by him in his very difficult enquiries, as well as for his dexterity and adroitness in conducting his experiments. We do regret, however, that he has been able to give us but negative or indirect proofs of the functions of the sympathetic nerve. The nature of the enquiry, the minute ramifications of the branches of this nerve, and the consequent, shall we say, impossibility of disengaging the several organs from their connexions with it, have served many others, and surely will not be denied to him, as an apology for the species of argument employed. Negative proofs are unsatisfactory in every department of science, more particularly in those branches of it where the senses are called in to decide.

---

We shall now present our readers with a succinct analysis of the work of the celebrated Lobstein, on the same subject. The work of this distinguished physiologist is divided into three parts or *sections*, the *first* containing the *anatomical* description of the sympathetic nerve; the various circumstances connected with the history of this nerve, the evolution of this nerve in the fœtus, and its intimate structure. *The second section* is purely *physiological*, and contains the various opinions regarding the use of the ganglions—the nature, use, and importance of the sympathetic nerve—observations on the sympathetic nerve of animals—an examination of the powers possessed by this nerve—and an enquiry into its several functions—concluded with some observations with respect to the mechanism by which this nerve performs its several functions. *The third section* is entirely *pathological*, and contains, first, the dynamic diseases of the sympathetic nerve, or functional affections of this nerve—consensual or sympathetic diseases, arising from an affection of this nerve—and, lastly, organic diseases of the sympathetic nerve. The anatomical description we shall altogether omit, as being entirely removed from the office of a periodical journal. The various conjectures regarding the functions of this nerve we shall not enumerate, for the reasons already assigned at the commencement of this article. We shall, therefore, proceed to lay before the reader an analysis of the pathological portion of this highly interesting work.

Physicians for a long time suspected that, in those nervous affections of the abdomen so often met with in practice, the ganglionic system was the seat of the disease, and, above all, that in hypochondriasis and hysteria, the abdominal nerves undergo some change. This suspicion is at the present day confirmed; and it it now established as a pathological dogma, that these diseases are to be referred exclusively to a simple neuralgia of the abdominal nerves. With respect to *hypochondriasis*, all the phenomena of this disease clearly prove it to be of a spasmodic nature, and that it consists in a *neuropathy* of the system of the ganglions. The sensation of unusual pressure in the præcordial region, after taking food, the difficult digestion of food, accompanied with flatus, the great degree of distress, palpitations of the heart, dizziness of the head, trembling of the limbs, &c. can have their seat and origin only in a disordered state of the nerves of the abdominal viscera. The vital action of the gastric organs, which, in the natural and healthy state, occasions no sensation whatever, now affects the brain after an unusual manner; the mind, influenced by this affection of the brain, forms an incorrect judgment of all that is going on in the abdomen. Hence it is, that the impression transmitted to the encephalon from the abdomen, through the branches of the nerves, excites sudden fright, dread of apoplexy or some other alarming disease, the patients being, at the same time, totally removed from any danger. Sometimes the disordered state of the abdominal nervous system, and the effects of this on the brain, so change the constitution of the mind, that, amidst the various disturbances of the fancy, the thoughts become altogether unconnected—symptomatic mania and real delirium are produced, which again disappear with the returning energy of the mind. Oftentimes the mind is so carried away, that one time the patient breaks forth into loud laughter from the slightest cause, returning soon after to a state of melancholy, and even to tears, without any apparent reason.

*Hysteria* indicates an affection of the uterine nervous system, which draws into sympathy with it the nerves of the epigastric organs, and particularly of the lungs; so that dyspnœa, and a sense of constriction of the trachea, is a common symptom of this disease. It sometimes assails the dominion of the brain also; and the augmented energy of the sympathetic nerve gives rise to considerable mental aberrations, and produces actions apparently spontaneous, which are, however, in reality, totally removed from the empire of the will.

In a slighter degree, the patients frequently complain of spasms, which commence in the abdominal region, extend imperceptibly through the breast, when they sometimes produce merely the sensation of a vibration and oscillation of the integuments and muscles of the head and face; sometimes they give rise to intolerably acute pains, seated sometimes in the sinciput, sometimes in the occiput—affecting one-half of the head in some, in some the entire head; sometimes these pains are permanently fixed in some one spot in the head, so small that it may be covered with the thumb, accompanied by a sense of cold; yielding to no remedies, save those which remove the inordinate and excessive sensibility of the abdominal viscera. Hence it is, that we frequently see pain, which was before most intense, become instantly more mild after only a single eructation, and dissipated in a few moments by means of a few drops of laudanum or a few grains of castor.

The regular progress of an hysteric attack was very strikingly marked in the case of a young woman, admitted into the hospital under the author's care. First spasms came on in the abdomen—then pain and a sense of inflation in the epigastrium, with a feeling of distress and efforts to vomit; then constriction of the lungs, dry cough, and palpitation of the heart; then attempts at deglutition, as if a lump of food was sticking in the pharynx, ready to be propelled into the stomach ; then complete aphonia. This series of symptoms attacked the patient repeatedly, so that one day one phenomenon appeared—another day the disease migrated, as it were, into another territory of the nerves. It originated in the hypogastric plexus ; from thence it ascended to the gastric and solar plexus ; from thence to the pulmonic and cardiac plexuses ; and from these to the laryngeal and recurrent plexuses. After this, the morbid affection descended by a retrograde movement, and in the same order in which it had ascended, into the pelvic organs, soon to retrace its pristine course.

The seat of *melancholy* and *mania* is placed, by the unanimous consent of medical men, in the abdominal viscera. These phenomena, formerly ascribed to obstructions of the viscera, particularly of the spleen, are now more correctly referred to alterations of the nerves. Neither is it vapours that ascend to the head, nor black bile that is secreted in these diseases ; but the solar plexus, or abdominal brain, re-acts on the cephalic brain, so as entirely to disturb its normal functions. This our author adduces as a proof of the close connexion between the abdominal and cephalic nervous centres. In the state of health, the harmony between these two centres is uninterrupted. In the same manner as the cephalic brain may be excited, and exalted to an extraordinary degree of power, so, also, the abdominal nervous centre sometimes rushes from its own peculiar sphere, which is to preside over the function of nutrition, and produces, in certain diseases, phenomena of the most extraordinary nature.

Our author here alludes to the powers of animal magnetism, by which, he says " it may be admitted as certain, that the abdominal nervous centre can be carried into a new sphere of action, the dignity of its functions exalted to the highest pitch, so that it takes on itself, as it were, the functions of the cerebrum itself, and displays extraordinary powers, of which we had before no conception, which we may, indeed, admire, but cannot explain." That our author is inclined somewhat to animal magnetism, would appear from the following passage :—

" I do not think that observations should be rejected, which seem to declare the existence of occult powers of nature ; and, in my opinion, it is unbecoming in the medical philosopher to treat with contempt phenomena, without previous investigation, solely because they are involved in obscurity, and do not square with received principles and vulgar notions. More extended experiments, conducted with an unbiassed mind, will probably teach us what we are to think of new, or rather renewed, enquiries regarding magnetism, and what improvement physiology may derive from them."

As a farther proof of the intimate connexion between the cephalic and abdominal brains, our author instances the fact, that the above diseases, scil. *melancholy* and *mania*, are frequently relieved, and occasionally cured, by the administration of medicines which irritate and stimulate the abdominal nerves. In this way may be accounted for the almost miraculous power of emetics, which merely excite nausea, the vast utility of which, in diseases of the mind, has been amply proved by experience. These medicines never act by their resolving powers, nor by evacuating sordes or morbid secretions in the alimentary canal, but as irritants applied to the gastric nerves, and then to the solar plexus, by which another mode of action is excited in these nerves; or at least, by reason of their intimate connexion with the brain, the morbid action of the latter organ is removed from it by revulsion, and directed to the abdomen on the principle of derivation.

*Lead colic* is seated unquestionably in the abdominal nerves, this being the only system to which we can refer the very acute pain of the intestines —the retrocession of the abdomen towards the spine—the constipation— tenesmus—suppression of urine—palpitations of the heart, &c. There is not a single symptom in this disease which may not be accounted for from the affection of this system of nerves.

Another disease which may be referred to irritation of the ganglionic system of nerves, is *angina pectoris*. This disease, according to our author, consists in an affection of the cardiac, and probably of the pulmonic nerves. It exerts its violence on the pectoral organs; constriction of the chest takes place, which gives rise to most acute pains; this, following a transverse direction, terminates at the arm, and excites in the patient inexpressible distress. The occasional causes of the disease, as well as its sudden attacks, prove it to be an idiopathic affection of the nerves. An objection may be made to this theory, derived from the bony incrustations so frequently found in the bodies of those who have died of this disease, at the roots of the aorta and pulmonary artery, in the semilunar valves, and often in the tunics of the coronary artery. Our author meets this objection by replying, that these ossifications are but effects, and not causes of the disease; inasmuch as they have frequently been found in these parts of the bodies of persons, who never had a symptom of the affection. To this he may have very safely added, that the symptoms have very frequently been observed in persons, in whom no such ossification was found after death. Our author conceives that we may readily understand the origin of these depositions, if we only recollect the dominion possessed by the nerves over the capillary vessels: the nervous branches act on the capillary system, and excite and exalt its normal functions, by which excitation the vessels are made to secrete a substance analogous to the tissue of those parts of the heart. Every pathologist knows how readily the fibrous tissue of arteries is penetrated by calcareous phosphate.

Another affection which our author refers to the same source is *incubus*, or *nightmare*. The feeling of oppression at the præcordia, with short and laborious breathing, experienced by persons when lying asleep on their back —the interruption of their voice and utterance—the hallucinations of the senses and reason—the attacks ceasing in a little time, and again returning —the fluttering of the heart, fright and perspiration—the great lassitude experienced after the paroxysm has terminated—the livid spots occasionally observed on the body, &c. indicate an idiopathic affection of the solar plexus, which, through the nervus vagus, transmits the most painful impressions to the brain, which, by means of the other anastomoses with almost all the nerves, produces unpleasant sensations in the limbs, and, finally, through its innumerable nervous ramifications, exerts its power on the capillary vessels, or, perhaps, excites an intestine motion in the blood itself. To this probably may be added, an electro-galvanic process, which, being spontaneously elicited, produces those shocks in the nerves and muscles, so well known to medical men.

Our author next proposes a conjecture with respect to the nature of intermittent fever, and suggests that it should be sought for in a perverted action of the abdominal system; and, in support of his conjectures, adduces some very plausible arguments; namely, that there very seldom occurs a case of this disease, in which the functions of the abdominal organs are not disturbed; that the paroxysm is often ushered in by vomiting; that the disease is frequently removed by means of purgatives alone; that a single emetic, given just before the paroxysm comes on, checks it, and sometimes cures the disease; from which it appears that another movement is impressed on the solar plexus, contrary to that which produced the fever; and, lastly, that the disease, when left to itself, or badly treated, produces infarctions of the abdominal viscera, such as induration of the liver, enlargement of the spleen, &c. by which a general morbid state is changed into a topical affection. Another argument in support of this view, is derived by our author from the regular rhythm observed in the recurrence of the paroxysms of intermittents, arising from their being rooted in the nervous system, the functions of which are performed periodically, according to a law assigned it by Nature.

These two systems of nerves are liable to their respective diseases. As the various convulsive diseases, epilepsy, tetanus, &c. affect the voluntary muscles, though no organic lesion can be detected in them, in the same manner the nerves of the thoracic and abdominal viscera may be irritated, without any change in them discoverable by the senses; as the perverted action of the *cephalic brain* acts with the utmost violence on the *abdominal brain*, so, vice versâ, the latter re-acts on it with no less severity, and destroys it; as, in fine, the cerebral system, stunned, as it were, by the violence of disease, closes the springs of life, there is reason to believe that the solar plexus does the very same in certain diseases. Sudden deaths are usually said to occur in three ways, through the *brain*, the *heart*, and the *lungs*. To this our author would add a fourth—namely, the *solar plexus*, the spring and centre of the abdominal nerves, a plexus which, seized on by any sudden commotion, may be affected with fatal paralysis, soon followed by death.

The author next introduces the subject of *sympathetic diseases*, the seat of which he places in the abdomen. Thus, *hemicrania* is frequently a concomitant of hypochondriasis and hysteria—*sympathetic headache* is often occasioned by obstruction of the bowels, which is proved by the great relief derived from the use of purgatives. Every medical man must be aware of the severe pain of head occasioned by worms in the intestines; worm fever is often known to assume all the symptoms of hydrocephalus, and all the bad symptoms are removed by the use of medicines calculated to improve that state of the abdominal organs.

*Agrypnia* or *sleeplessness* is often sympathetic, and may be classed under the head of affections of the abdominal nerves. This will appear evident to any one who considers the many irritations, which, arising from the abdomen, increase the nervous action, accelerate the motion of the blood towards the head, and interfere with sleep. Hence it is, that persons who have crammed themselves at supper oftentimes awake after a short sleep, and are unable to sleep again.

*Sympathetic affections of the eyes and nose* are too well known to require much to be said on them. How often do we see ophthalmia cured by directing attention to the state of the abdominal viscera. Vitiated secretions in the abdomen, which irritate its nerves, have often occasioned dilatation of the pupil, nyctalopia, hemeralopia, amaurosis, &c. Our author, after detailing various other local affections, which have their primary cause in the abdominal nervous centre, now shews how an inordinate or morbid state of these nerves may gradually and imperceptibly draw the several organs within their morbid sphere, and ultimately produce diseases in which real change of structure may be recognized.

The various depressing passions are capable of altering the natural and physiological state of the abdominal nerves.* Spasms are thus produced, by which the action of the vessels is disturbed ; the necessary consequence of this is congestion of the fluids in the capillaries, the motion of the blood being retarded ; thence infarction of the vessels, obstructions of the viscera, and genuine organic diseases. To this source of disease, our author refers aneurisms of the heart and great vessels. An attentive observation of disease has convinced our author, that spasmodic symptoms always, and for a long time, precede organic change.

1st. Every one knows that, in affections of the head, convulsive motions of the muscles of the face, grinding of the teeth, strabismus, &c. indicate serious lesion of the encephalon. These symptoms should be classed among the precursors of hydrocephalus, inasmuch as they are frequently combated with success, which scarcely ever happens, if there be a collection of water already in the ventricles. The perverted action of the brain is not confined within the cavity of the cranium, but extends to distant organs, by which means organic diseases are produced. Inflammation and suppuration of the liver, after lesions of the brain, have no doubt been preceded by a dynamic state in the nervous apparatus of the liver. Various hypotheses have been devised, in order to explain the connexion between the encephalon and hepatic system, none of which approach probability. Our author would prefer to appeal to nervous communication, particularly that between the right nervus vagus and the solar plexus, through which the brain is associated with the right semilunar ganglion, from which the posterior hepatic nerves directly arise.

2d. Aneurisms of the heart are preceded by nervous irritation, particularly when they arise from an arthritic taint, and are formed slowly. The

---

* A case somewhat in point will be found in the last number of this Journal, p. 184. The remote cause of this disease, in the individual referred to, was *mental anxiety*—this produced disorded functions of the stomach and bowels : the sequences, nay, the consequences, are so graphically detailed by the attending physician, Dr. J. Johnson, that we shall refer the reader to the article itself.—REV.

heart for a long time palpitates, and suffers tremors and nervous oscillations, before it is affected with real dilatation.

3d. Various are the morbid affections which usher in morbid changes in the lungs. Patients are for many years subject to spasmodic attacks, which are ultimately succeeded by ossification in the trunks of the arteries arising from the heart, as in angina. Our author has seen cases of phthisis, which, at the commencement, presented merely symptoms of abdominal neuralgia. The lungs, in these persons, seemed to be absolutely exempt from disease, so that one would have said that the general and nervous affections were suddenly changed into an organic lesion.

4th. In abdominal diseases, the nerves have been affected sympathetically before they produce the organic lesions in the viscera to which they belong. The author has seen instances of induration of the pylorus, which came on after hypochondriasis of long standing.

The author has seen all the signs of inflammation in the sympathetic nerve, which inclines him to think that other organic changes may be found in it, if proper attention were used. He relates the case of a woman who died of exhaustion, occasioned by long-continued and obstinate vomiting, which nothing could stop. At the post-mortem examination, he found the semilunar ganglia of an intensely red colour, which was evident and genuine inflammation. This woman had been, for a long time, labouring under hypochondriasis, excruciating headaches, and raving in her sleep. She had had all these symptoms from puberty, had married at the age of 42, had become pregnant, which latter circumstance, added to domestic misfortunes, aggravated all the preceding symptoms, and ultimately produced the vomiting of which she died.

The value of the practical observations contained in the work of which we have given this analysis, will, we trust, be a sufficient excuse with our readers for the length of it. We said, at the commencement of this analysis, that, to the practical physician, there is no department of physiology and pathology in which he can feel more deeply interested, than in the knowledge of the functions of the sympathetic nerve. Whether the authors whose names are prefixed to this article have done any thing to extend our knowledge of it, we shall leave to the reader's judgment. No one certainly will deny the value of the hints contained in that part of M. Lobstein's work which has been now presented to him.

---

Illustrations of the Comparative Anatomy of the Nervous System—Part I. By *Joseph Swan.* London: Longman and Co. 1835.

